# Identification and verification of the key genes involved in gallbladder cancer

**DOI:** 10.3389/fimmu.2025.1643366

**Published:** 2025-09-04

**Authors:** Jie Tang, Hanxu Zhou, Miao Lu, Dengyi Cao, Yun Zhang, Shaobo Zhou

**Affiliations:** ^1^ The Affiliated Wuxi People’s Hospital of Nanjing Medical University, Wuxi, Jiangsu, China; ^2^ Wuxi Medical Center, Nanjing Medical University, Wuxi, Jiangsu, China; ^3^ Wuxi People’s Hospital, Wuxi, Jiangsu, China; ^4^ General Surgery, The Second Affiliated Hospital of Bengbu Medical College, Bengbu, Anhui, China; ^5^ General Surgery, Shenzhen Yantian District People’s Hospital, Shenzhen, Guangdong, China

**Keywords:** gallbladder cancer, GPR64, exosome, biomarker, bioinformatics

## Abstract

**Background:**

Gallbladder cancer (GBC) is a highly aggressive malignancy of the biliary tract. It often lacks distinct symptoms in its early stages, and no specific biomarkers have yet been identified for its diagnosis.

**Objective:**

To identify key genes involved in GBC pathogenesis using public databases and bioinformatics analysis and validate these findings experimentally, providing a foundation for developing potential GBC biomarkers.

**Methods:**

Analysis of GBC-related data from the Gene Expression Omnibus database revealed that G protein-coupled receptor 64 (GPR64) was differentially expressed in GBC. GPR64 expression in GBC-SD and NOZ cells was modulated using lentiviral transfection. Functional assays assessed cancer-related phenotypes, while apoptosis was measured using flow cytometry. Xenograft models in nude mice were established with cell lines overexpressing GPR64.

**Results:**

GPR64 expression was reduced in GBC. Its overexpression suppressed GBC cell invasion, migration, and proliferation, and induced apoptosis. *In vivo* findings were consistent with *in vitro* results.

**Conclusion:**

GPR64 plays a critical role in GBC pathogenesis and may serve as a promising biomarker for its diagnosis and treatment.

## Introduction

Gallbladder cancer (GBC) is a highly malignant type of cholangiocarcinoma. The incidence and mortality of cholangiocarcinoma are rising in several regions across the world (1), albeit their corresponding rates vary based on factors such as the anatomical site or gender. For instance, intrahepatic and extrahepatic cholangiocarcinoma are more frequent in men, whereas GBC is more common in women (1, 2). Moreover, both the incidence and mortality rates have been found to increase with age (3, 4). Because GBC has an insidious onset and lacks reliable detection methods, early diagnosis is difficult. Most cases are discovered incidentally, contributing to poor prognosis (5, 6). Although chronic inflammation is a key contributor, the exact mechanisms underlying GBC remain unclear (7). Surgical resection remains the most effective treatment (8). However, many patients present at advanced stages and are not surgical candidates (9, 10). Thus, identifying sensitive diagnostic biomarkers is essential for early detection, improved treatment options, and the development of targeted therapies.

G protein-coupled receptor 64 (GPR64) is classified as a G protein-coupled receptor (GPCR) family, which is also known as adhesion GPCRG2 or human epididymis-specific protein 6, belonging to the GPCR family (11, 12). It is expressed mainly in the proximal epididymis and excretory tubules, which are involved in sperm maturation (13), and is considered a transmembrane protein specific to the epididymis (13, 14). GPCRs play critical roles in cancer progression (15, 16). For example, SMPD1 and GPR64 are downstream targets of EWS-FLI1, and the SMPD1–ceramide–GPR64 axis promotes Ewing’s sarcoma growth (17). In endometrial adenocarcinoma, GPR64 is expressed at low levels and acts as a tumor suppressor (18). Gene profiling of ovarian endometrioid adenocarcinoma has identified GPR64 as a novel target in the β-catenin/T-cell factor-signaling pathway (19). GPCRs regulate numerous physiological processes and are key drug targets in diseases such as obesity, psychiatric disorders, and cancer (13). However, GPR64’s role in GBC is not well defined.

Exosomes are extracellular vesicles approximately 30 – 200 nm in size. They facilitate substance transfer between cells and carry proteins, lipids, RNA, and other molecules (20). Exosome-mediated pathways influence the immune microenvironment, tissue stability, cancer, and infections (21, 22). Therefore, further investigation of the association between the exosome-associated gene *GPR64* and GBC pathogenesis may help identify useful biomarkers and improve the outcomes for patients with GBC.

## Materials and methods

### Main reagents and chemicals

Human GBC cell lines GBC-SD and NOZ (sourced from The Chinese Academy of Sciences and The iCell Bioscience Inc., respectively) were cultured in Dulbecco’s Modified Eagle’s Medium (DMEM; California, USA) supplemented with 1% antibiotics and 10% fetal bovine serum; GIBCO, Grand Island, NY, USA. Nude mice (BALB/c, age: 6 – 8 weeks) were obtained from the Cavens Model Animal Research Company (Suzhou, China). A lentivirus vector was procured from the Shanghai Jikai Gene Company (Shanghai, China). The GPR64 monoclonal antibody (host: mouse; isotype: IgG1) was obtained from Wuhan Sanying Biotechnology Company (Wuhan, China).

### Data acquisition and processing

All animal experiments were performed in adherence to the guidelines and protocols of the China Animal Protection Association. The ethics committee of Bengbu Medical University provided its ethical approval (Ethics Approval Letter (2024) 377). Relevant datasets (i.e., GSE238179 and GSE255497) were downloaded from the Gene Expression Omnibus (GEO) database and organized and visualized using Perl and R software. The data was obtained in the following order: Search keywords (Biliary Tract Cancer:1631), Top Organisms (Homo sapiens: 1625), Entry type (Series: 76), Study type (Expression profiling by array: 21). The first two items that best matched the search criteria were selected, that is GSE238179:8 and GSE255497:32. The corresponding clinical information is given in **Supplementary File 1** and **Supplementary File 2**. Exosome-related genes were sourced from the https://www.genecards.org/website (861 websites) and the related literature (18 sources).

### Identification of differentially expressed genes

To reduce the data bias between the two datasets (i.e., GSE238179 and GSE255497), principal component analysis (PCA) was performed on the expression matrix before and after eliminating the batch effects. The filtering condition was set to logFC absolute value >1 and *P* < 0.05, and the differential analysis and visualization were conducted using relevant R packages (limma, dplyr, pheatmap, and ggplot2).

### DEGs enrichment analysis

To further explore the potential biological functions of DEGs in the pathogenesis of GBC, we analyzed the differential genes of GBC by Gene Ontology (GO), Kyoto Encyclopedia of Genes and Genomes (KEGG), and Gene Set Enrichment Analysis (GSEA) methods for analysis. KEGG and GSEA enrichment analysis revealed the enrichment of the related pathways and genes.

### Construction and verification of the diagnostic model

Least Absolute Shrinkage and Selection Operator (LASSO) regression analysis was performed on all the DEGs of GBC, and the results of LASSO regression analysis were regarded as model genes. Then, the highest accuracy and the lowest error rate were determined by the Support Vector Machine (SVM) algorithm and further applied to identify the DEGs. The key genes were identified in the LASSO regression model and SVM analysis. Finally, a receiver operating characteristic (ROC) curve was drawn for the key genes to evaluate the accuracy of the model.

### Analysis of the key gene immune microenvironment

To explore the function and role of DEGs in the immune microenvironment of GBC, the GSVA package and GSEA Base package of R software were used to immunologically score the DEGs of GBC. The key gene scores were extracted for analysis, and the correlation heat map was visualized with the R pheatmap package to analyze the correlation results between immune cells and the key differential genes.

### Lentiviral transfection

A cell suspension (1 mL; density of 3 – 5 x 10^4^/mL) was mixed with 3 mL of DMEM and incubated in a 6-well plate for 16 – 24 h. A certain amount of virus and infection enhancer was then added in accordance with the MOI and virus titer of the cells specified in the instructions. The mixture was cultured for 12 – 16 h, and the medium was refreshed to continue culturing for the determination of the change time based on cell morphology. After approximately 3 days of incubation, the cells were transfected under a fluorescence microscope, puromycin was used to screen the uninfected cells, and the virus volume was calculated as follows: (MOI × cell count)/virus titer. (GPR64-OE: GPR64 Overexpression, GPR64-NC: GPR64 Negative Control). The RT-PCR primer sequence used in the study was GPR64 F:CAGGCGTCAAACCCCAGAG, GPR64 R:CCAGTTAAGGTGCCATTCGTTAT. GAPDH F: CAGGAGGCATTGCTGATGAT,GAPDH R: GAAGGCTGGGGCTCATTT.

### Subcutaneous tumorigenesis assay

Six different groups (namely, GBC-SD-GPR64-OE, GBC-SD-GPR64-NC, GBC-SD, NOZ-GPR64-OE, NOZ-GPR64-NC, and NOZ) of cell suspensions were prepared at the same density and injected into the upper-right abdomen of nude mice (n = 30, Density: 5 × 10^7^/mL). The tumor volume was measured once every 5 days (volume [mm^3^] = 0.5 × width^2^ × length). On day 30, all mice were euthanized, and their tumor tissues were harvested for subsequent analyses.

### Western blotting

Proteins were extracted and quantified in accordance with the instructions provided with the RIPA lysis solution (Beyotime, Jiangsu, China) and BCA assay kit (Jiangsu, China), respectively, followed by loading, electrophoresis, membrane transfer, blocking with milk, treatment with primary antibody, washing, treatment with secondary antibody, and washing. Finally, ECL was used to expose the images in a gel imaging system (Bio-Rad, USA). Other antibodies used included B-cell lymphoma-2 (Bcl-2) antibody/neural cadherin (N-cadherin) antibody/Bcl-2-associated X protein (Bax) antibody/vimentin antibody/cysteine-dependent aspartate-specific protease-3 (caspase-3) antibody/goat anti-rabbit IgG (primary antibody dilution ratio of 1:1000, secondary antibody dilution ratio of 1:10000; Jiangsu Qinke Biological Research Center Co., Ltd., Jiangsu, China).

### Immunohistochemistry

Paraffin sections of subcutaneous tumors obtained from the experimental mice were dewaxed and hydrated, followed by blocking with goat serum (Beyotime) for 30 min and incubation with the primary antibody in a wet box at 4 °C overnight. Next, the sections were washed and incubated with the secondary antibody. Color was developed using DAB (Beyotime) color developing solution after washing; brown and yellow staining indicated a positive result. After washing, the nucleus was stained and then gently dehydrated, meticulously sealed, and observed.

### Hematoxylin-eosin staining

The paraffin sections of the murine subcutaneous tumors were dewaxed and hydrated. Hematoxylin staining of the cell nucleus and differentiation with 1% hydrochloride alcohol for 10 s were then performed, after which the slices were treated with 0.6% ammonia water and rinsed with clean water. These sections were then stained with eosin for 5 min after gradient ethanol dehydration and sealed using neutral gum gel. The stained sections were then imaged under a microscope, and the observations were recorded.

### Transwell migration and invasion assays

The experiment was conducted in accordance with the detailed steps described in the Transwell assay manual (23). Briefly, the cells were seeded into a 6-well plate chamber without and with matrix gel and incubated in serum-free culture medium. To this, 10% fetal bovine serum was added into the lower chamber medium and allowed to culture for 1 day in the cell incubator, followed by gentle washing with phosphate-buffered saline (PBS), fixing with polyformaldehyde, staining, and, finally, photography.

### Wound healing assay

The cell suspension was inoculated into a 6-well plate after grouping. When the cells matured, a 200-µL pipette was used to scratch the cells and observe and photograph them at the same position under a microscope at 0, 24, and 48 h timepoints.

### Cell colony formation

Cells showing an adherence rate of >90% were digested, centrifuged, resuspended, and counted. Then, 1 mL of the cell suspension was added to each well of a 6-well plate at a density of 1000 cells/mL and placed in a CO_2_ incubator. The medium was changed according to predefined conditions. After 2 weeks of culture period, by when the colonies were formed, the cells were fixed and stained.

### Flow cytometry analysis

After the cells reached approximately 90% confluence, they were subjected to trypsin digestion and collection. The cells were washed in pre-cooled PBS twice, and a cell suspension was prepared. For cell staining, 5 µL of Annexin V-APC was added to the cell suspension and mixed well, to which 10 µL of PI was added and mixed. After incubation at room temperature for approximately 15 min, flow cytometry was performed, and the results were recorded.

### Statistical analysis

All experiments were repeated more than three times. The data were analyzed using GraphPad Prism and expressed as the mean ± standard deviation. Statistical analyses were performed using the *t*-test, one-way analysis of variance (ANOVA), Mann–Whitney U-test, and Ruscall–Wallis test. Statistical significance was set to *P* < 0.05.

## Results

### Differential analysis of GBC genes

**Figure 1:**
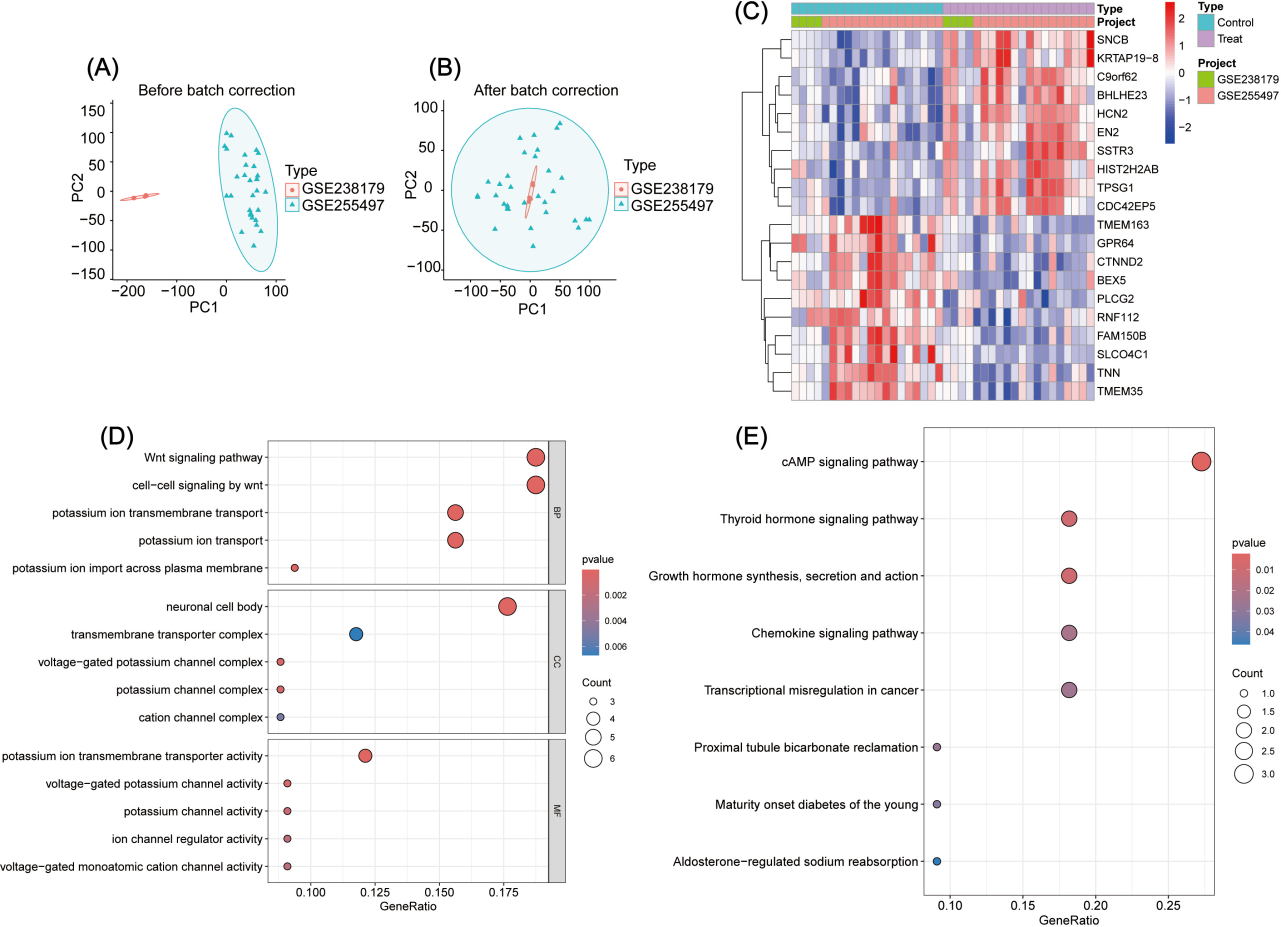
**(A, B)** Analysis of intersection genes between GSE238179 and GSE255497: A, Before data correction; B, After data correction. **(C)** Intersection gene difference analysis: Display of the heatmap of the top ten genes with the most significant differences. **(D)** GO enrichment analysis of DEGs. **(E)** KEGG enrichment analysis of DEGs.

Relevant datasets (GSE238179, GSE255497) were downloaded from the GEO database. Using Perl and R, the data were processed, corrected, and visualized to identify intersecting genes (**Figures 1A, B**). Further differential analysis revealed 16 upregulated and 27 downregulated genes (**Supplementary File 3**). The top 10 genes in each category are displayed in the heatmap (**Figure 1C**).

### DEGs enrichment analysis

**Figure 2:**
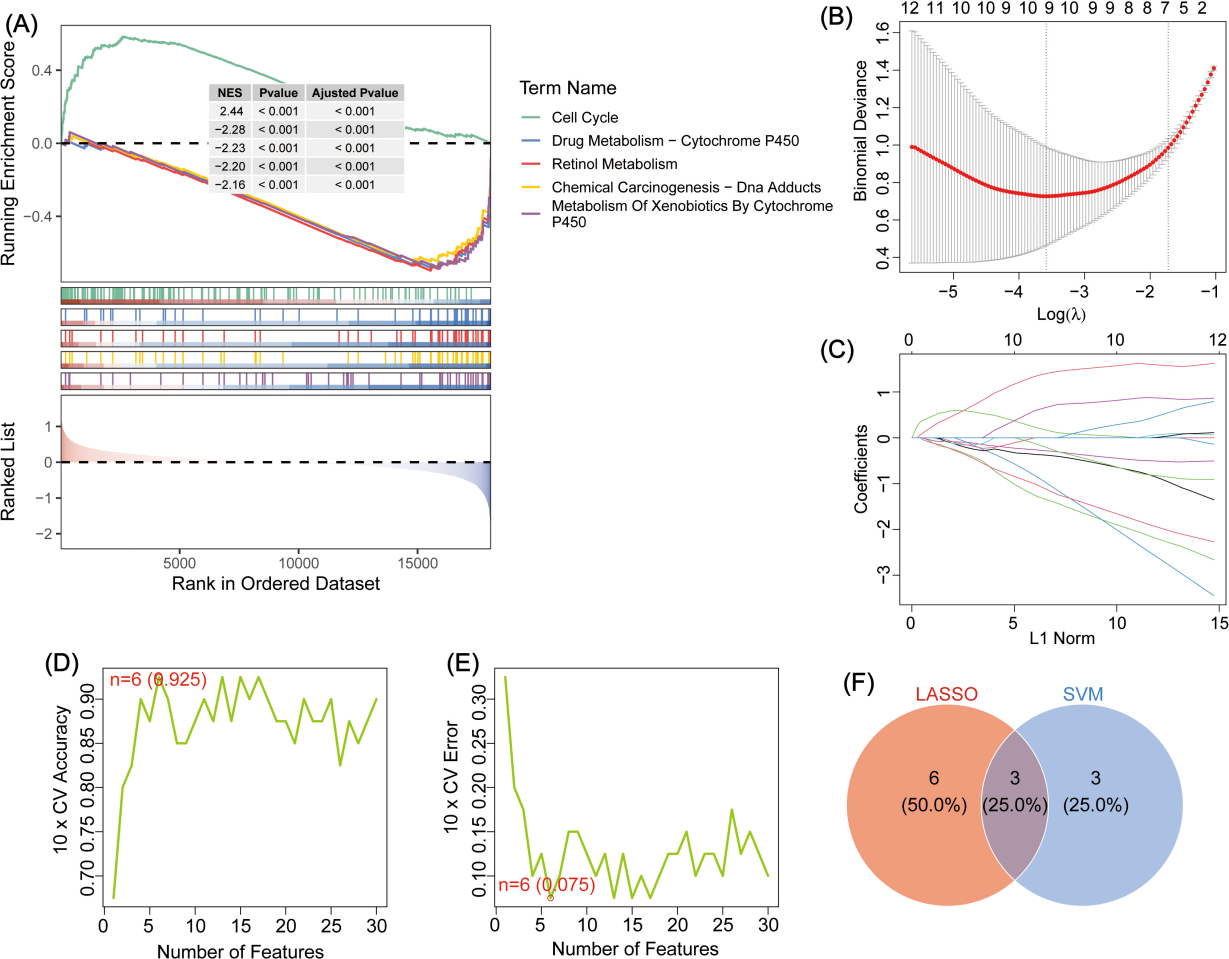
**(A)** GSEA enrichment analysis of DEGs, showing the top five. **(B)** Schematic diagram depicting LASSO regression diagnostic model for DEGs in GBC. **(C)** Variable trajectory map of the LASSO diagnostic model. **(D)** SVM algorithm to identify the number of genes with the highest accuracy. **(E)** The SVM algorithm was used to identify the number of genes with the lowest error rate. **(F)** Venn map depicting the intersection between LASSO and SVM genes; the intersecting genes: TMEM163, GPR64, and RNF112.

To explore the biological functions of DEGs in GBC pathogenesis, GO, KEGG, and GSEA enrichment analyses were performed (**Supplementarys Files 4**–**6**). GO analysis showed enrichment in the following: Biological process: Wnt-signaling pathway, cell–cell signaling by Wnt, and potassium ion transmembrane transport; Cellular component: neuronal cell body, transmembrane transporter complex, and voltage-gated potassium channel complex; and Molecular function: potassium ion transmembrane transporter activity, voltage-gated potassium channel activity, and potassium channel activity (**Figure 1D**). KEGG analysis revealed the top three pathways as cAMP signaling, thyroid hormone signaling, and growth hormone synthesis, secretion, and action (**Figure 1E**). GSEA identified the top five pathways as Cell Cycle, Drug Metabolism – Cytochrome P450, Retinol Metabolism, Chemical Carcinogenesis – DNA Adducts, and Metabolism of Xenobiotics by Cytochrome P450 (**Figure 2A**).

### Screening and validation of model genes

**Figure 3:**
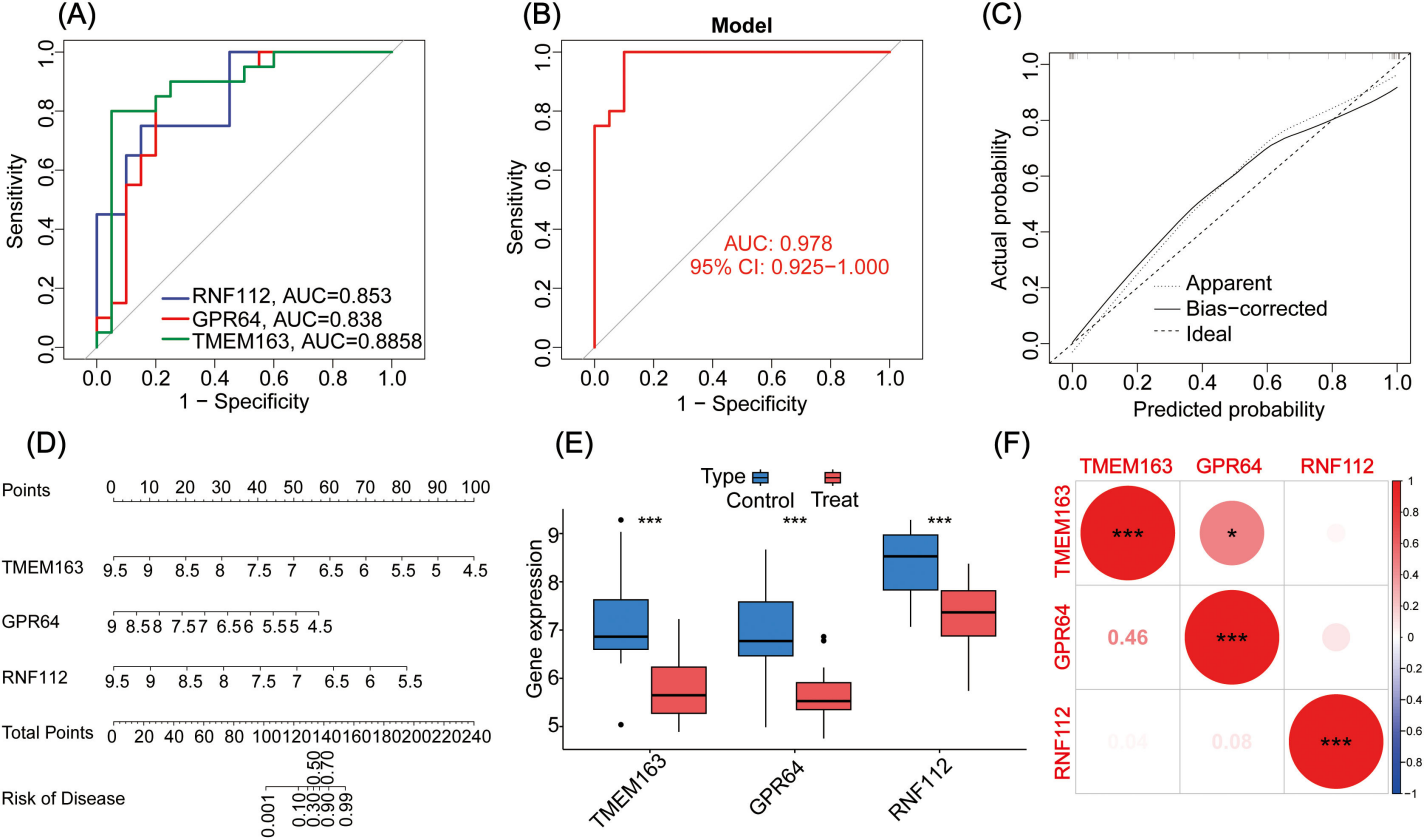
**(A)** The ROC curves for three genes (i.e., TMEM163, GPR64, and RNF112). **(B)** The ROC curve analysis of the key gene models. **(C)** Calibration curve of the GBC diagnostic model gene. **(D)** Nomogram of genes for the GBC diagnosis model. **(E)** Analysis of differential expression of the key genes in the GBC control group and treatment group. **(F)** Correlational heat map of the key genes in GBC. *P < 0.05, ***P < 0.001.

To identify key genes involved in GBC pathogenesis, LASSO and SVM were used for dual screening (**Figures 2B–E**), yielding three crucial DEGs: TMEM163, GPR64, and RNF112 (**Figure 2F**). ROC curve analysis was conducted to assess diagnostic performance: RNF112 (AUC = 0.853), GPR64 (AUC = 0.838), and TMEM163 (AUC = 0.8858) (**Figure 3A**). The combined AUC for all three genes was 0.978 (**Figure 3B**), indicating high diagnostic accuracy for GBC.

A nomogram was constructed to evaluate the correlation between gene expression and GBC risk. The results showed that lower gene expression corresponded to higher risk scores (**Figure 3D**). The calibration curve confirmed the predictive accuracy of the three-gene model (**Figure 3C**).

Expression levels of key genes were visualized in control and treatment groups, clearly showing that all three function as tumor suppressor genes (**Figure 3E**). Additionally, their interrelationships were analyzed (**Figure 3F**).

### Analysis of the immune microenvironment of key genes

**Figure 4:**
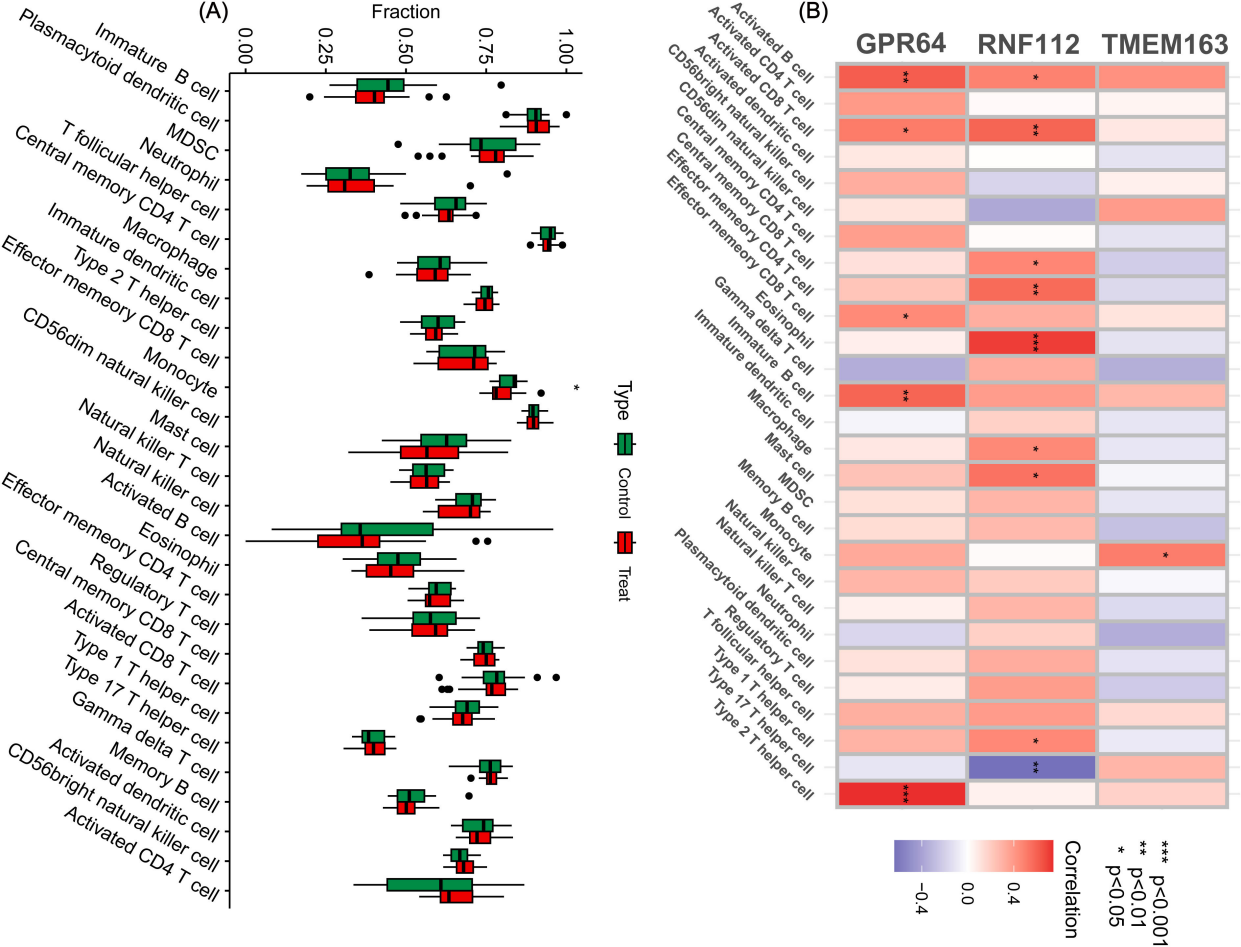
Immune cell infiltration analysis. **(A)** By analyzing the key genes through ssGSEA, a box plot of 28 immune cells was displayed between the control and treatment groups. **(B)** Heat map of immune cell correlation analysis of the key genes. Red represents a positive correlation, whereas blue represents a negative correlation. The darker the color, the stronger the correlation. (^*^ *P* < 0.05, ^**^ *P* < 0.01, ^***^ *P* < 0.001).

To further investigate the impact of key genes on the immune microenvironment in GBC, immune infiltration was assessed using ssGSEA. The results showed that these genes were associated with monocytes (**Figure 4A**). A heat map generated in R revealed correlations between key genes and immune cell infiltration. GPR64 was linked to activated B cells, activated CD8 T cells, effector memory CD8 T cells, immature B cells, and type 2 T helper cells. RNF112 was associated with activated B cells, activated CD8 T cells, central memory CD8 T cells, effector memory CD4 T cells, eosinophils, macrophages, mast cells, and type 1 and type 17 T helper cells. TMEM163 was associated only with monocytes (**Figure 4B**).

### GPR64 is associated with exosomes and modulates GBC progression

**Figure 5:**
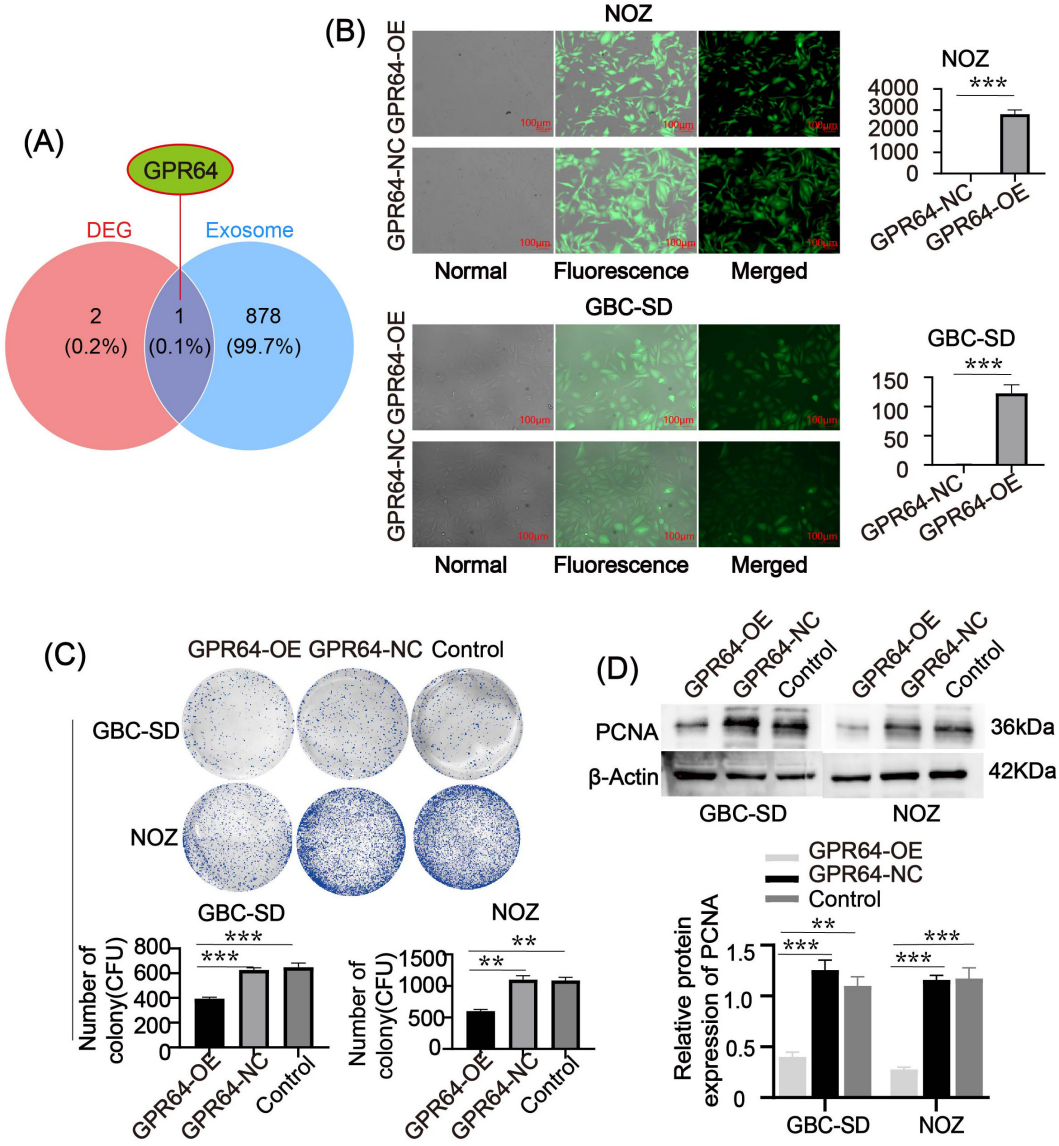
**(A)** Venn diagram depicting the intersection between key genes and exosome genes. GPR64 is an intersecting gene. **(B)** Fluorescence diagram of two GBC cell lines (GBC-SD, NOZ) after lentivirus transfection. After successful transfection, the expression of GPR64 in GBC cell lines (GBC-SD, NOZ) significantly increased. **(C)** The colony-formation experiment depicted that the number of cell colonies formed by the GPR64-OE group was markedly lower than in the GPR64-NC group and control group (Cell lines: GBC-SD, NOZ). **(D)** The WB revealed that the expression of proliferation-related protein PCNA in the GPR64-OE group was lower than that in the GPR64-NC group and control group (Cell lines: GBC-SD and NOZ). **P < 0.01, ***P < 0.001.

We combined the key genes with exosome-related genes (exosome genes:https://www.genecards.org/) to investigate their possible roles in GBC pathogenesis. GPR64 was identified as an exosome-associated gene (**Figure 5A**). Lentiviral transfection was used to overexpress GPR64 in GBC cell lines (GBC-SD, NOZ), and transfection efficiency was confirmed by RT-PCR (**Figure 5B**). GPR64 expression in the overexpression group was significantly higher than in the negative control group.

Colony formation assays showed that the number of colonies in the GPR64-OE group was significantly lower than in the GPR64-NC and Control groups (**Figure 5C**). WB analysis indicated reduced levels of the proliferation-related protein PCNA in the GPR64-OE group compared to the GPR64-NC and Control groups (**Figure 5D**).

**Figure 6:**
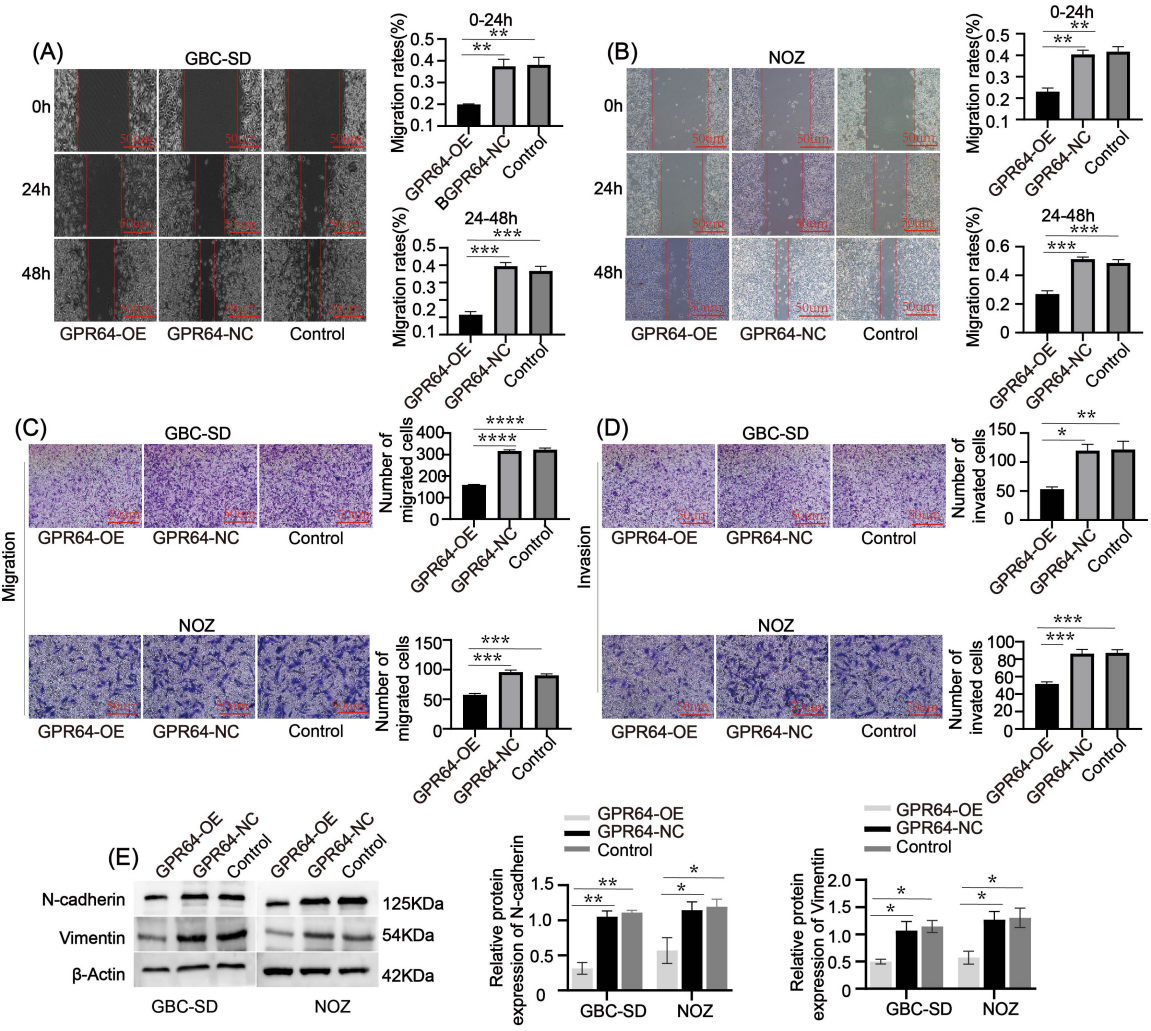
**(A, B)** The wound-healing assay indicated that the wound-healing rate of the GPR64-NC and control groups was faster than that of the GPR64-OE group (Cell lines: GBC-SD and NOZ) at 24 and 48 h, respectively. **(C, D)** The Transwell assay indicated that the total number of cells passing through the chamber in the GPR64-OE group was significantly lower than that in the GPR64-NC and control groups (Cell lines: GBC-SD and NOZ). **(E)** The WB experiment revealed that the expressions of migration-related protein vimentin/N-cadherin in the GPR64-OE group were significantly lower than those in the GPR64-NC and control groups (Cell lines: GBC-SD and NOZ). *P < 0.05, **P < 0.01, ***P < 0.001, ****P < 0.0001.

Additionally, the wound healing rate at 0 – 24 h and 24 – 48 h was significantly lower in the GPR64-OE group than in the GPR64-NC and Control groups (**Figures 6A, B**). Transwell assays showed reduced cell migration and invasion in the GPR64-OE group compared to the corresponding Control group (**Figures 6C, D**). WB results further demonstrated that the expression of migration-related proteins Vimentin and N-cadherin was significantly lower in the GPR64-OE group than in the GPR64-NC and Control groups (**Figure 6E**) (all *P* < 0.05). These findings suggest that GPR64 overexpression inhibits the migration, invasion, and proliferation of GBC-SD and NOZ cells.

### Relationship between GPR64 and apoptosis of GBC cells

**Figure 7:**
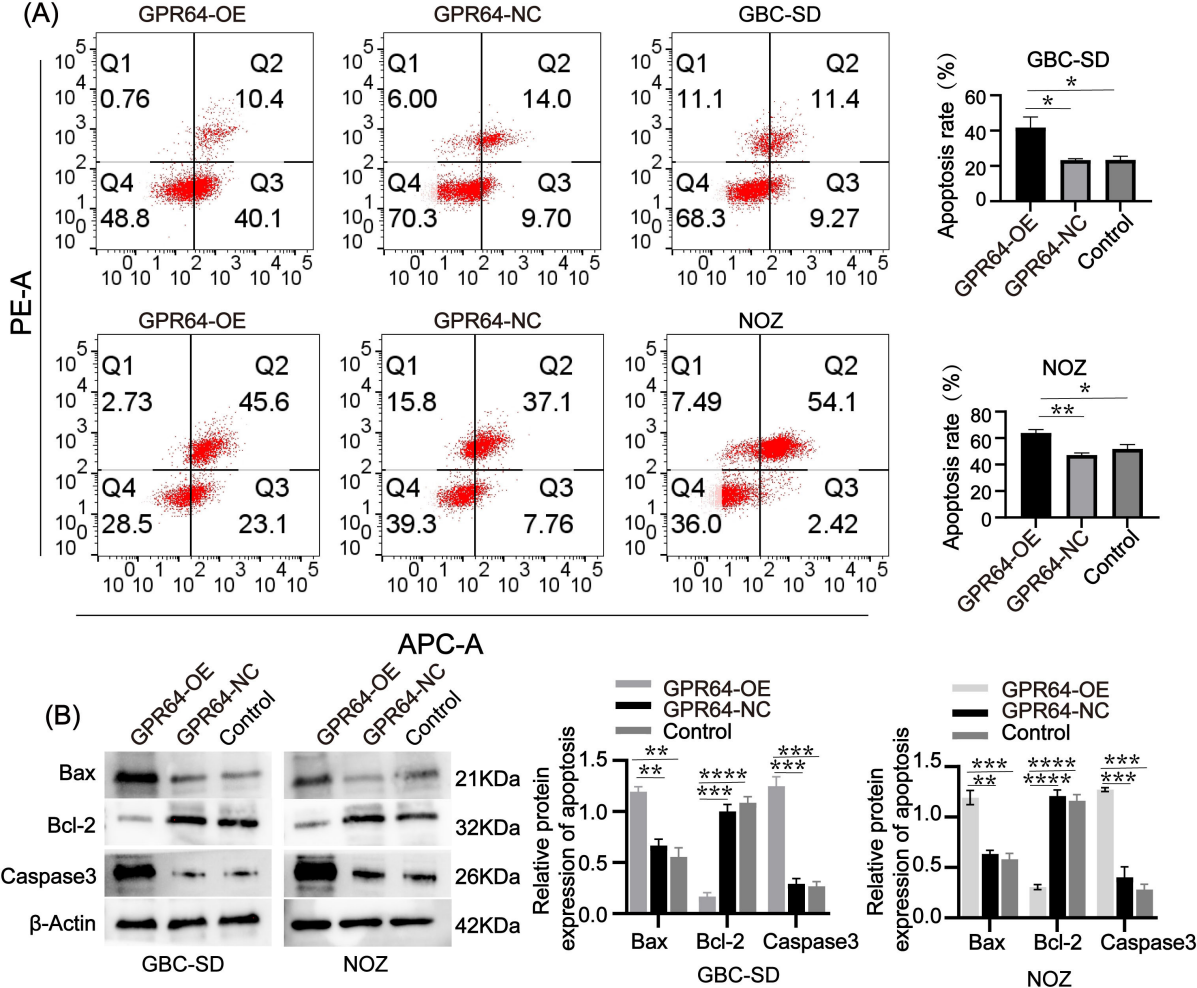
**(A)** Flow cytometry after transfection revealed that the apoptosis rate of the GPR64-OE group was significantly higher than that of the GPR64-NC and control groups (Cell lines: GBC-SD and NOZ). **(B)** The WB revealed that the expression of apoptosis-related proteins (Bax/Caspase-3) in the GPR64-OE group was significantly higher than that in the GPR64-NC and control groups (Cell lines: GBC-SD and NOZ). Meanwhile, when compared with the GPR64-NC and control groups, the level of anti-apoptotic protein (Bcl-2) was significantly reduced in the GPR64-OE group (Cell lines: GBC-SD and NOZ). *P* < 0.05). *P < 0.05, **P < 0.01, ***P < 0.001, ****P < 0.0001.

Flow cytometry analysis revealed that the apoptosis rate in the GBC-SD-GPR64-OE and NOZ-GPR64-OE groups was higher than that in the GBC-SD-GPR64-NC, NOZ-GPR64-NC, and Control groups (**Figure 7A**). WB analysis showed that pro-apoptotic proteins Bax and Caspase-3 were significantly higher in the GBC-SD-GPR64-OE and NOZ-GPR64-OE groups than in the GBC-SD-GPR64-NC, NOZ-GPR64-NC, and Control groups, while the anti-apoptotic protein Bcl-2 was significantly lower in the GBC-SD-GPR64-OE and NOZ-GPR64-OE groups than in the GBC-SD-GPR64-NC, NOZ-GPR64-NC, and Control groups (**Figure 7B**). These results indicate that GPR64 overexpression significantly enhances apoptosis in GBC cell lines (GBC-SD and NOZ).

### GPR64 overexpression inhibited subcutaneous tumor growth and angiogenesis in nude mice

**Figure 8:**
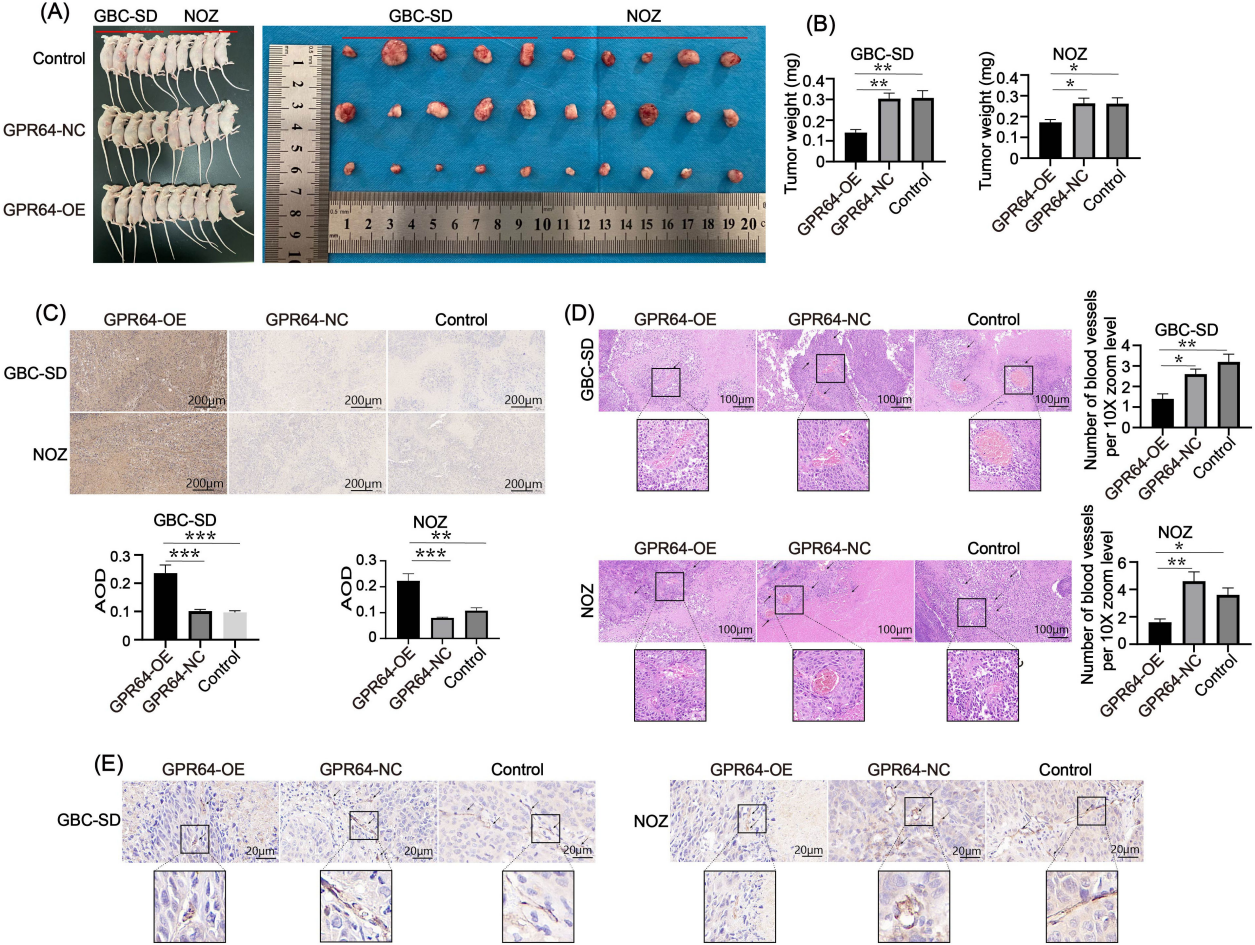
**(A)** Subcutaneous tumor formation in the three groups of nude mice using two cell lines (Cell lines: GBC-SD and NOZ). **(B)** On the 30^th^ day, the tumor weight of the GPR64-NC and control groups was heavier than that of the GPR64-OE group. **(C)** Immunohistochemical analysis of the GPR64 tumor tissues in nude mice showed that AOD of the GPR64-OE group was significantly higher than that in the GPR64-NC and control groups (Cell lines: GBC-SD and NOZ). **(D)** H&E staining revealed that the number of newly formed blood vessels in the GPR64-OE group was significantly smaller than that in the corresponding GPR64-NC and control groups (Cell lines: GBC-SD and NOZ) (10X magnification). **(E)** Immunohistochemical analysis of vascular marker CD31 revealed that the positive areas in the GPR64-OE group were fewer than those in the corresponding GPR64-NC and control groups (Cell lines: GBC-SD and NOZ) (40X magnification). *P* < 0.05. *P < 0.05, **P < 0.01, ***P < 0.001.

Subcutaneous tumors were collected from three groups of nude mice (**Figure 8A**). On day 30, tumors in the GBC-SD-GPR64-NC, NOZ-GPR64-NC, and Control groups weighed significantly more than those in the GBC-SD-GPR64-OE and NOZ-GPR64-OE groups (**Figure 8B**), suggesting that GPR64 overexpression effectively suppresses tumorigenicity in GBC cells. Immunohistochemical staining of tumors from both cell lines (GBC-SD, NOZ) showed that the average optical density of GPR64-positive areas was higher in the GPR64-OE group than in the GPR64-NC and Control groups (**Figure 8C**).

Moreover, neovascularization in tumors in the GPR64-OE group was significantly lower than that in the GPR64-NC and Control groups (**Figures 8D, E**), suggesting that GPR64 exerts its tumor-suppressive effect by modulating tumor angiogenesis.

Overall, GPR64 significantly inhibits GBC progression by affecting the migration, invasion, proliferation, apoptosis, and angiogenesis of GBC cells.

## Discussion

The integration of bioinformatics into medicine has become increasingly comprehensive, encompassing genomics, epigenomics, transcriptomics, proteomics, metabolomics, and other fields (24). These tools allow researchers to extract meaningful insights from large-scale data and apply them to disease research and analysis (25). Bioinformatics has already contributed significantly to solving diagnostic and therapeutic challenges in complex diseases. For instance, Gong Liuyun et al. demonstrated aspirin’s therapeutic potential on specific targets in small cell lung cancer through bioinformatics analysis (26). Lu Xiaoqing et al. identified novel biomarkers and therapeutic targets for gastric cancer using differential gene analysis and core gene screening (27). Wenxue Zhang et al. analyzed GEO and TCGA datasets and found that HIST1H2BH was upregulated in multiple myeloma, suggesting it as a critical gene for diagnosis and therapy (28). These cases highlight the practical value of bioinformatics in clinical research. However, the scope of bioinformatics extends beyond these examples, and its proper application in medicine is crucial for improving disease diagnosis and treatment.

GBC, though rare, is highly aggressive and accounts for 80%–95% of all biliary tract malignancies (29). Histological subtypes include adenocarcinoma, adenosquamous carcinoma, and undifferentiated carcinoma, with adenocarcinoma being the most prevalent (7, 30). However, survival rates do not differ substantially across subtypes (8), largely because GBC is typically asymptomatic in its early stages. Patients often present with abdominal pain only during intermediate or advanced disease, delaying clinical intervention (31). At this point, even with a confirmed diagnosis, the optimal treatment window is often missed (32, 33). While modern cancer therapies—including neoadjuvant therapy, adjuvant therapy, radiotherapy, and immunotherapy—have demonstrated success in many malignancies (34, 35), their impact on GBC remains limited (36). Currently, surgical resection is the only effective intervention for GBC (37). Therefore, discovering reliable early diagnostic biomarkers is vital to enable timely surgery and improve patient outcomes.

Although the precise pathogenesis of GBC remains undefined, prolonged chronic inflammation is widely considered a key contributing factor (7). With the advancement of multi-omics technologies, inflammation and the immune microenvironment have been increasingly recognized as pivotal factors in GBC development, providing novel insights into its diagnosis and treatment strategies (38). During cancer progression, genomic stability is compromised by a range of intrinsic and extrinsic influences, particularly those involving immune system dynamics (39, 40). Immunotherapy has emerged as a promising approach enhancing the host immune system’s ability to target tumors and offering new avenues for cancer management (41, 42). Approaches such as immune checkpoint inhibitors, cancer vaccines, and adoptive cell therapies have demonstrated encouraging clinical outcomes (43). For instance, mRNA-based platforms can be engineered to deliver modified antigens to antigen-presenting cells, thereby stimulating T lymphocytes to initiate robust anti-tumor responses (44). Additionally, engineered nano systems have been developed to activate the cGAS/STING pathway, effectively inhibiting tumor growth and recurrence by reversing immunosuppressive state within the tumor microenvironment (45). This therapeutic strategy leverages the cGAS–STING signaling cascade, which detects aberrant cytoplasmic DNA and induces inflammatory responses, thereby reshaping the tumor immune landscape (46).

The application of systems biology, particularly through big data computation and advanced bioinformatics techniques, has transformed medical research. One of its most impactful contributions is the identification of molecular biomarkers, enabling precise and targeted disease therapies (47). In this study, we employed Perl and R programming to perform repeated analyses of GEO datasets (48, 49). By integrating two GEO datasets, conducting differential gene analysis, and building predictive models, we identified GPR64 as a potential exosome-associated tumor suppressor gene implicated in GBC pathogenesis. Moreover, Shi Lei et al. reported that GPR64 was encapsulated in exosomes and released into the tumor microenvironment, enhancing the invasive and metastatic abilities of cancer cells by activating the NF-κB-pathway and upregulating the levels of MMP9 and IL - 8 (50). This approach provided a direction for studying GPR64 in GBC. The present research revealed that GPR64 was associated with activated B-cells, activated CD8 T-cells, effector memory CD8 T-cells, immature B-cells, and type 2 T-helper cells in the pathogenesis of GBC. Considering these associations, we hypothesized that interaction of GPR64 with activated B-cells and type 2 T-helper cells may influence the immune cell activity, consequently affecting GBC cells’ response to immunological attacks. Moreover, when GPR64 interacts with activated CD8 T-cells and effector memory CD8 T-cells, it may impair their cytotoxic functions, thereby reducing the effectiveness of immune cells in eliminating cancer cells.

GPR64, as a potential biomarker in GBC patients, may help improve the diagnostic accuracy, identify high-risk groups, and predict the effectiveness of immunotherapy. As such, it has important translational significance in the early diagnosis, in treatment decision-making, and in the discovery of new targets, which may improve the survival rate and quality of life of GBC patients. However, this study primarily relies on analyses of publicly available databases and computational simulations, which may be constrained by limitations such as insufficient sample size and limited population diversity (51, 52). While bioinformatics excels in large-scale data interpretation and pattern recognition, it typically provides only a preliminary framework for investigating disease mechanisms (25). For instance, gene expression profiling can identify differential gene activity across disease states, but it does not fully capture the complexity of gene–gene interactions or elucidate their functional roles in disease progression (25, 53). Although both *in vivo* and *in vitro* validation were performed in this study, no validation was performed with human tissues. Therefore, these results alone do not adequately elucidate the molecular landscape of GBC. In the future, we plan to expand on the insights generated by this analysis, with a focus on the immune microenvironment in GBC. Although the precise mechanisms by which GPR64 regulates apoptosis, cell migration, and immune cell infiltration at the signaling pathway level remain unclear, potential modes of action can be inferred from studies on other GPR family members such as GPR132 and GPR65. For instance, GPR64 may influence apoptosis by modulating specific signaling cascades, either through activation or inhibition. It could regulate cell migration by affecting cytoskeletal dynamics, the expression of adhesion molecules, or the secretion of chemokines. Additionally, GPR64 may impact immune cell infiltration by altering immune cell activation, proliferation, migratory capacity, and chemotactic behavior.

Specifically, in the future, we aim to investigate how immune components influence GBC progression and how GPR64 interacts with the immune microenvironment, as well as the contribution of exosomes in immune regulation. Although this direction presents substantial challenges, it offers a critical theoretical basis for identifying novel biomarkers and improving the diagnostic and therapeutic strategies for GBC.

## Conclusion

GPR64 is a key gene involved in the pathogenesis of GBC. Continued investigation into its function holds significant promise for advancing biomarker discovery and improving diagnosis and treatment in GBC.

## Data Availability

The original contributions presented in the study are included in the article/**Supplementary Material**, further inquiries can be directed to the corresponding authors.
