# Supplementary figures and images for: Identification and verification of the key genes involved in gallbladder cancer
Source: Front Immunol. 2025 Sep 4;16:1643366. doi: 10.3389/fimmu.2025.1643366 (PMC12443716; doi:10.3389/fimmu.2025.1643366)

Figure 5D

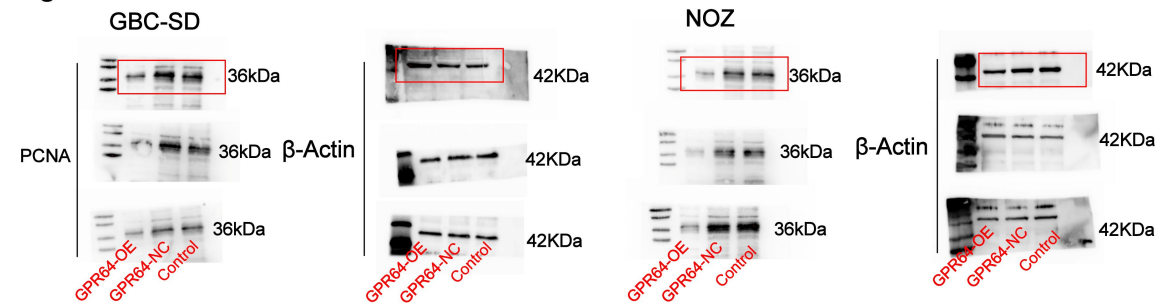

Figure 6E

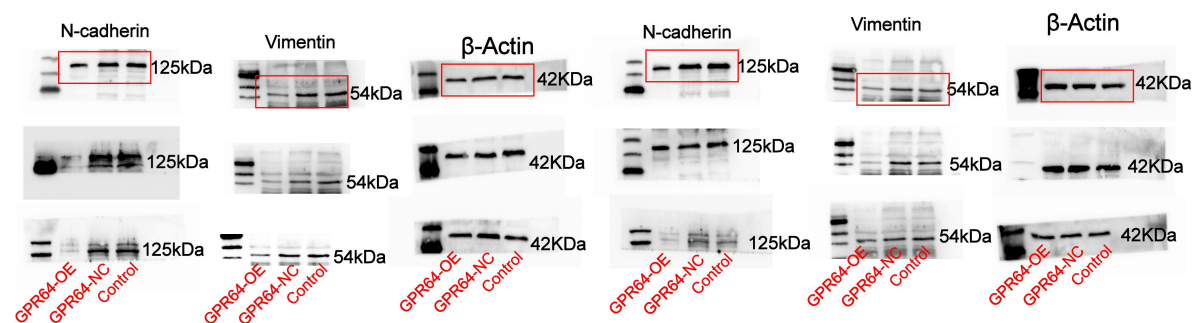

Figure 7B

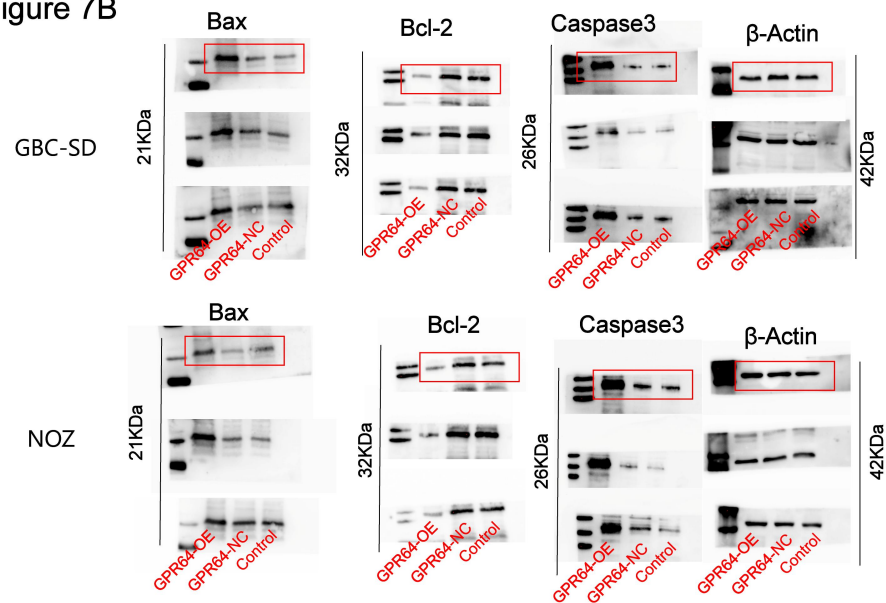

Supplement: Supplementary file 1 [file DataSheet1.pdf]
